# Identification and validation of genes related to stem cells and telomere maintenance mechanisms as biomarkers for breast cancer

**DOI:** 10.3389/fimmu.2025.1618193

**Published:** 2025-07-01

**Authors:** Shuang Zhen, Lifeng Huang, Qiannan Zhu, Rui Chen, Jue Wang, Xiaoming Zha

**Affiliations:** Department of Breast Surgery, Department of General Surgery, The First Affiliated Hospital with Nanjing Medical University, Nanjing, China

**Keywords:** breast cancer, telomere maintenance mechanism, stem cell, whole transcriptome, single-cell RNA sequencing, biomarkers

## Abstract

**Background:**

Stem cell-related genes (SCRGs) and telomere maintenance mechanism-related genes (TMMRGs) are pivotal in breast cancer (BC) pathogenesis by facilitating tumor cell proliferation and self-renewal. This study employed integrated transcriptomic and single-cell RNA sequencing (scRNA-seq) analyses to investigate SCRGs and TMMRGs as potential biomarkers for BC and to elucidate their underlying cellular mechanisms.

**Methods:**

Total RNA was extracted from eight BC tumor samples and eight matched adjacent non-tumorous tissues. Differential expression profiling, protein-protein interaction (PPI) network construction, and Molecular Complex Detection (MCODE) were conducted. Biomarker candidates were identified using the least absolute shrinkage and selection operator (LASSO) algorithm, followed by pathway enrichment and immunological analyses. Publicly available scRNA-seq datasets were utilized to delineate BC cell types, with emphasis on cellular subsets exhibiting differential biomarker expression. Heterogeneity, communication, and pseudo-temporal analyses of key cells were examined. Biomarker expression was further validated by reverse transcription-quantitative polymerase chain reaction (RT-qPCR).

**Results:**

JUN, NFKB1, and SP1 were significantly downregulated in BC, potentially modulating disease progression through mechanisms involving extracellular matrix (ECM) remodeling, intracellular signaling, oxidative stress response, and translational regulation. Activated B cells and natural killer (NK) cells demonstrated elevated infiltration levels, accompanied by increased expression of immune checkpoint molecules CD200, CD274, TIGIT, TNFRSF25, and TNFSF15. Nine distinct cellular lineages were annotated, among which mesenchymal cells exhibited pronounced biomarker expression differences and enhanced differentiation potential, designating them as key cellular mediators. Interactions between mesenchymal subpopulations (MSC1, MSC2, MSC3) and other cell types were markedly reduced in BC, despite an overall expansion in mesenchymal cell numbers during disease progression. MSC1 emerged as the predominant subtype. RT-qPCR analyses corroborated the downregulation of JUN, NFKB1, and SP1 in BC tissues.

**Conclusion:**

JUN, NFKB1, and SP1 were identified as potential biomarkers for BC. These findings highlight the critical role of mesenchymal cells in tumor biology and suggest potential therapeutic targets.

## 1 Introduction

Breast cancer (BC) remains one of the most prevalent malignancies among women globally, with incidence rates continuing to escalate and posing a significant public health burden (1). Established risk factors—including age, sex, ethnicity, family history, genetic mutations, timing of menarche and menopause, and hormone replacement therapy—contribute to the diagnostic and therapeutic complexity of BC, adversely impacting patient survival (1–4). Although therapeutic advances have been made, five-year survival outcomes are still largely contingent upon the timeliness and accuracy of disease detection (5). The multifaceted nature of BC and its unpredictable clinical course highlight the urgent need for novel biomarkers to enhance early diagnosis, clarify underlying mechanisms, and support individualized treatment approaches, ultimately improving therapeutic outcomes and patient well-being (4, 6, 7). Consequently, intensified efforts in biomarker research are essential not only for advancing the biological understanding of BC but also for informing preventive, diagnostic, and therapeutic innovations—crucial for optimizing global healthcare resource allocation.

Telomeres, composed of repetitive nucleotide sequences at chromosomal termini, preserve genomic stability by preventing degradation and end-to-end fusion during mitosis. Progressive telomere shortening with successive cell divisions ultimately triggers replicative senescence or apoptosis upon reaching a critical threshold (8). Studies have shown that the shortening of telomeres is closely related to the senescence of immune cells, which can lead to the exhaustion of T cell function and the decline of proliferation ability, thereby weakening the immune response of the body (9–11). Telomere maintenance mechanisms (TMM), predominantly regulated by telomerase (12), counteract this attrition, enabling sustained cellular proliferation (13).The maintenance of telomere length is crucial for the normal function of cells, and telomere dysfunction is associated with various diseases (14). In cancer research, the activation of TMM is strongly linked to the occurrence and development of tumors. For instance, TMM activation in malignant neuroblastoma is closely associated with high-risk diseases and poor prognosis (15). Studies on hepatocellular carcinoma (HCC) have also shown that telomere maintenance plays a key role in the occurrence mechanism of HCC and helps define the clinical characteristics of patients (16). Stem cells, defined by their capacity for self-renewal and multilineage differentiation, are pivotal in tissue homeostasis, regeneration, and development (17, 18). Due to the unique immune escape mechanism of stem cells, they are crucial in the occurrence and development of tumor and immune therapy resistance (19–21). For example, tumor stem cells can down-regulate the expression of major histocompatibility complex class I (MHC-I) molecules, therefore reducing antigen presentation and making it difficult for cytotoxic T lymphocytes to recognize and kill tumor cells (22). The interplay between TMM, stem cells, and BC is particularly significant (23, 24). Dysregulated TMM activity, frequently observed in malignancies including BC, facilitates the bypass of senescence and confers unlimited replicative potential to tumor cells (25). Numerous studies have shown that telomerase reactivation is a common strategy employed by cancer cells to stabilize telomeres and circumvent programmed cell death. Additionally, cancer stem cells—a distinct subset with tumor-initiating and self-renewing properties—exhibit robust telomere maintenance activity and engage in oncogenic signaling cascades that drive tumor progression and metastasis (26, 27). TMM supports the proliferative and regenerative capacity of these cells, thereby contributing to oncogenesis and therapeutic resistance. Despite substantial evidence linking TMM and stem cells to BC pathogenesis, the precise molecular pathways mediating their effects remain incompletely elucidated (28).

Single-cell RNA sequencing (scRNA-seq) can reveal the dynamic changes, functional status and intercellular interactions of tumor-infiltrating immune cells at the single-cell level, providing key insights in clarifying the mechanism of anti-tumor immune responses (29, 30). At present, many studies have been conducted on tumors through scRNA-seq sharing. Liu et al. revealed the immunosuppressive effect of APOE+ macrophages in immune checkpoint inhibitor therapy through scRNA-seq analysis (31). Xie et al. elucidated the landscape of BC brain metastases through scRNA-seq analysis and identified ILF2 as a potential therapeutic target (32). Meanwhile, they also clarified the contribution of intercellular communication of the tumor microenvironment cells guided by histone chaperones to BC metastasis through scRNA-seq analysis (33). These studies indicate that single-cell sequencing technology can systematically analyze the heterogeneity of the tumor immune microenvironment, providing pivotal data for revealing treatment resistance mechanisms and discovering new therapy targets.

BC is orchestrated by intricate genetic and cellular networks, wherein stem cell-related genes (SCRGs) and TMM-related genes (TMMRGs) play pivotal roles in sustaining tumor cell renewal and unchecked growth. Elucidating the function of these genes at both the transcriptomic and single-cell resolution offers a promising strategy for uncovering actionable biomarkers and therapeutic targets. This study integrated transcriptomic profiling and scRNA-seq to characterize SCRGs and TMMRGs in BC. Tumor and matched adjacent normal tissue samples from patients with BC, alongside publicly available scRNA-seq datasets, were analyzed to assess gene expression patterns and functional relevance. By delineating key biomarkers and mapping their expression across diverse cellular subsets, this investigation aims to clarify their roles in BC progression. The resulting insights enhance the molecular understanding of BC and inform the development of precision therapies aimed at improving clinical outcomes.

## 2 Materials and methods

### 2.1 Data source

Tumor tissue samples were collected from eight patients with BC at The First Affiliated Hospital of Nanjing Medical University, with matched paracancerous tissues from eight healthy individuals serving as controls. All specimens underwent RNA-seq and stringent quality control (QC) to form the whole transcriptome RNA-seq dataset. The study was approved by the Ethics Committee of The First Affiliated Hospital of Nanjing Medical University, and informed consent was obtained from all participants.

To enhance analytical robustness, additional BC-related datasets were sourced from public repositories. Specifically, RNA-seq data comprising 1,082 BC tumor samples and 113 paracancerous samples were retrieved from The Cancer Genome Atlas (TCGA) database (https://portal.gdc.cancer.gov/) for expression validation. Concurrently, the scRNA-seq dataset GSE245601, based on the GPL18573 platform, was downloaded from the Gene Expression Omnibus (GEO) database (https://www.ncbi.nlm.nih.gov/gds), including 10 BC tumor samples and 2 normal tissue samples for cellular-level expression analysis (34).

**Figure 1:**
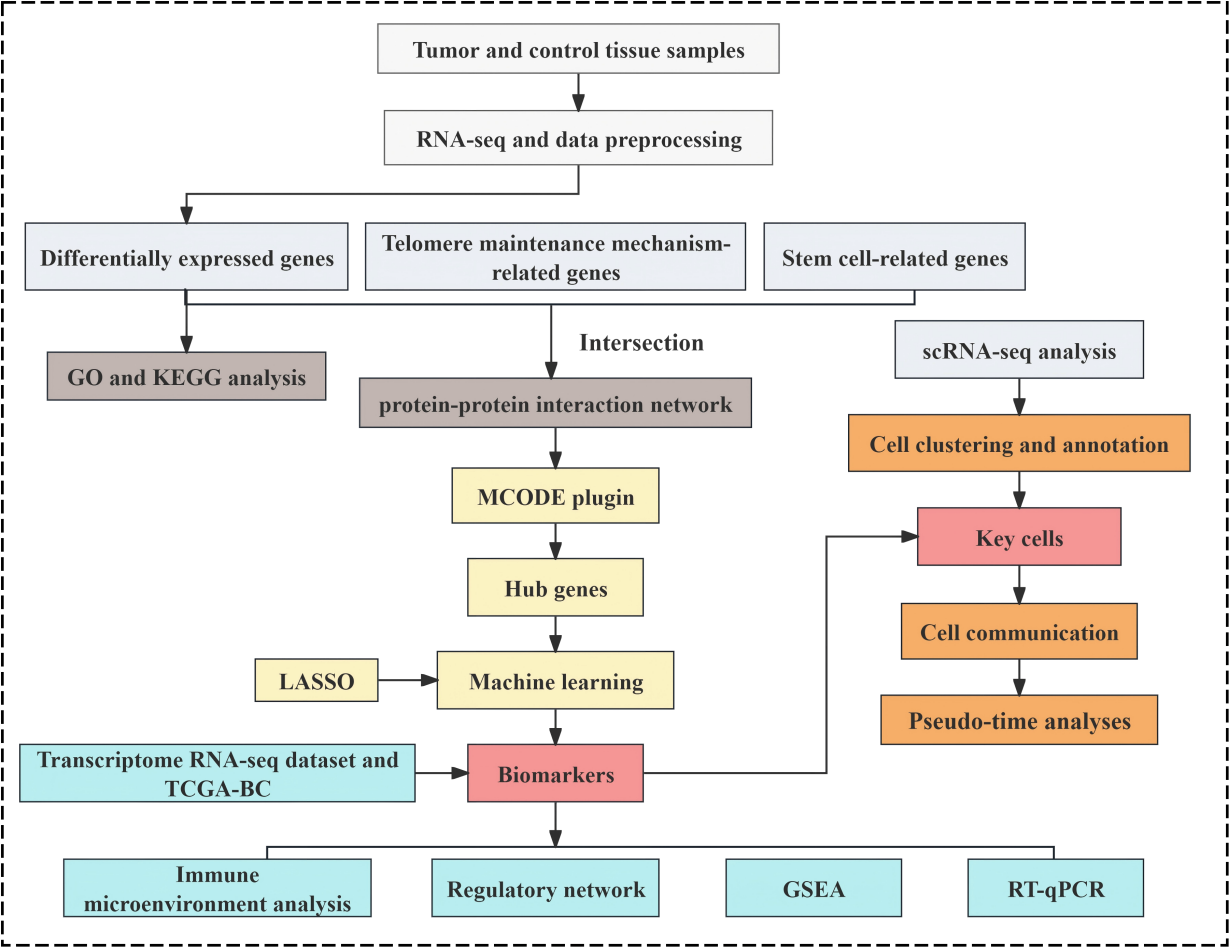
The analysis flowchart of this study.

Stem cell-associated gene data were curated by referencing established literature (35), and 26 stem cell gene sets were extracted from the StemChecker database (http://stemchecker.sysbiolab.eu/), yielding 4,419 SCRGs. Additionally, 218 TMMRGs were incorporated based on published literature (13). The overall analytical workflow of this study was illustrated in **Figure 1**.

### 2.2 RNA-seq and data preprocessing

Total RNA from the 8 tumor and 8 paracancerous tissue samples was isolated using TRIzol reagent (Invitrogen, CA, USA). RNA integrity and concentration were evaluated using a NanoDrop ND-1000 spectrophotometer (Wilmington, DE, USA) and an Agilent Bioanalyzer 2100 system (Agilent, CA, USA). Sequencing libraries were prepared with the Hieff NGS Ultifaillumina Dual-mode mRNA Library Prep Kit to generate inserts of 300 ± 50 bp, followed by high-throughput paired-end sequencing (PE150) on the Illumina NovaSeq 6000 platform.

Raw sequencing data in FASTQ format underwent QC using fastp software (v0.23.4) (36) with default parameters, including adapter trimming, deduplication, and removal of low-quality reads. Cleaned reads were aligned to the human reference genome (GRCh38) using HISAT2 (v2.2.1) (37), and alignment outputs were stored in BAM format. Transcript assembly and quantification were performed using StringTie (v2.2.0) (38), and expression levels were normalized to FPKM, calculated as:


FPKM=total exon fragments/(mapped reads in millions×exon length in kilobases).

### 2.3 Construction of differential expression analysis and enrichment analysis

Differential expression analysis between tumor and normal tissues was conducted on the transcriptome-wide RNA-seq data using the DESeq2 package (v1.38.0) (39). Differentially expressed genes (DEGs) were defined by |log_2_ fold change (FC)| > 0.5 and P < 0.05. Visualization of DEGs was performed using a volcano plot and heatmap, generated with ggplot2 (v3.3.6) (40) and ComplexHeatmap (v2.14.0) (41), respectively. Gene Ontology (GO) and Kyoto Encyclopedia of Genes and Genomes (KEGG) enrichment analyses were performed using the clusterProfiler package (v4.7.1.3) (42), with significance set at P < 0.05. GO terms were classified into three categories: biological processes (BPs), molecular functions (MFs), and cellular components (CCs).

### 2.4 Identifying hub genes through protein-protein interaction network visualization

The intersection of DEGs, SCRGs, and TMMRGs was identified using the VennDiagram package (v1.7.1) (43) to isolate candidate genes implicated in both stemness and telomere regulation within the context of BC. These candidate genes were subsequently analyzed using the Search Tool for the Retrieval of Interacting Genes (STRING) database (http://string-db.org) with a confidence threshold set at > 0.7, and a protein–protein interaction (PPI) network was generated to elucidate potential molecular interactions at the protein level. The Molecular Complex Detection (MCODE) plugin was used to select the highest-scoring cluster and identify hub genes. A PPI network was subsequently constructed for these hub genes. Both networks were generated using Cytoscape software (v 3.9.1) (44).

### 2.5 Combining machine learning algorithms and expression profiling to screen biomarkers

To assess the diagnostic potential of the hub genes, Least Absolute Shrinkage and Selection Operator (LASSO) regression analysis was performed using the glmnet package (v4.1.4) (45), configured with the ‘binomial’ family parameter. The optimal model was established at the point of minimal model error and the lowest lambda value, at which the genes as candidate biomarkers were selected for expression analysis. These genes were then subjected to expression analysis in both the in-house whole transcriptome RNA-seq dataset and the TCGA-BC dataset, comparing tumor and normal tissues. Expression differences were visualized *via* box plots generated with the ggplot2 package, and genes exhibiting consistent and statistically significant differential expression across both datasets (P < 0.05) were selected as biomarkers.

### 2.6 Gene set enrichment analysis

To investigate the functional relevance of these biomarkers, enrichment analyses were conducted on the whole transcriptome dataset. Using the Molecular Signatures Database (MSigDB, https://www.gsea-msigdb.org/gsea/msigdb), the curated gene set collection c2.cp.v2023.2.Hs.symbols.gmt was employed. For each biomarker, Spearman correlation coefficients were computed with all other genes, and genes were ranked in descending order of correlation. GSEA was performed using the clusterProfiler package, with statistical thresholds set at P < 0.05 and False Discovery Rate (FDR) < 0.05. The top five significantly enriched pathways per biomarker were visualized using the enrichplot package (v1.18.4) (46), ranked by enrichment significance.

### 2.7 Analyzing immune microenvironment differences and immune checkpoint expression profiling

To further explore the differences in the immune microenvironment between patients with BC and normal individuals, a single-sample GSEA (ssGSEA) approach from the GSVA package (v1.42.0) (47) was applied to estimate the infiltration levels of 28 predefined immune cell types (48) within each sample of the whole transcriptome dataset. Comparative analysis revealed statistically significant differences in immune cell abundance between tumor and normal samples (P < 0.05). Additionally, a set of 40 immune checkpoints was compiled from previously published literature (49), and differences in the expression of these checkpoints between tumor and normal samples were analyzed within the same dataset (P < 0.05).

### 2.8 Drug prediction analysis and construction of regulatory network

Drug–biomarker interactions were identified using the Drug Gene Interaction Database (DGIDB, https://dgidb.org), with the top five candidates ranked by interaction score. The resulting drug–biomarker network was visualized using Cytoscape.

To investigate the underlying molecular mechanisms of the biomarkers, the DESeq2 package was employed to analyze differentially expressed miRNAs and lncRNAs (DE-miRNAs and DE-lncRNAs) from a whole transcriptome RNA-seq dataset, comparing tumor and normal tissues under the criteria of P < 0.05 and |log_2_FC| > 0.5. Volcano plots were generated to depict the distribution of DE-miRNAs and DE-lncRNAs.

Candidate miRNAs targeting the biomarkers were predicted *via* miRDB (https://mirdb.org/) and TargetScan (https://www.targetscan.org/) using a prediction score threshold of 500,000. Correspondingly, target lncRNAs for these miRNAs were identified through starBase (http://starbase.sysu.edu.cn/index.php), filtered by a clipExpNum > 4.

Subsequent analysis involved intersecting DE-miRNAs with the predicted miRNA targets and DE-lncRNAs with the predicted lncRNA targets to isolate key regulatory miRNAs and lncRNAs. A lncRNA–miRNA–mRNA regulatory network was then constructed in Cytoscape to elucidate the multifaceted regulatory landscape influencing biomarker expression.

### 2.9 ScRNA-seq analysis

To investigate the cellular mechanisms underlying BC and to characterize biomarker heterogeneity at the single-cell level, comprehensive analyses were conducted using the GSE245601 dataset. QC, clustering, and annotation were performed with the Seurat package (v5.0.1) (50). Key quality metrics—including gene count, cell count, and mitochondrial gene percentage—were calculated. Cells with fewer than 200 genes and genes covered by fewer than three cells were filtered out, as were cells expressing either less than 200 or more than 5000 genes. Genes expressed in fewer than three cells or with total counts below 500 or above 20,000 were filtered out. Mitochondrial content was capped at 10%. Distributions of nFeature_RNA, nCount_RNA, and percent.mt before and after filtering were visualized, and only cells meeting all criteria were retained for downstream analysis. Subsequently, data normalization was applied, and the top 2,000 highly variable genes were selected using the vst method. Principal component analysis (PCA) was conducted to evaluate variance across components, with results visualized *via* a PCA elbow plot. Statistically significant principal components, identified using the JackStrawPlot function based on gene-level P-values, were selected for further analysis. Unsupervised clustering was performed using the FindNeighbors and FindClusters functions, and clusters were visualized using Uniform Manifold Approximation and Projection (UMAP) at a resolution of 0.4. Cell type annotation was based on canonical marker genes reported in prior research (34), and a bubble plot was generated to illustrate the specificity of these markers across identified cell populations. In addition, the NF-κB activation, stemness-related signatures, and ERK-JUN signaling pathway scores of the annotated cell types were evaluated using the AddModuleScore method.

### 2.10 Identification and heterogeneity analysis of key cells

In the scRNA-seq analysis, the AddModuleScore function from the Seurat package was utilized to compute enrichment scores of specific gene sets for each sample in the GSE245601 dataset, thereby assessing metabolic pathway activity across cell populations. A curated background gene set, encompassing candidate genes associated with both stem cells and TMM in BC, served as the basis for this scoring. Scores were calculated across annotated cell types in tumor and normal tissues, and cell populations demonstrating significant differences (P < 0.05) were designated as candidate key cells. Subsequent single-cell-level analysis examined the expression profiles of biomarkers within these candidate key cells across tumor and normal tissues. Cells exhibiting consistent and significant differential expression across all biomarkers (P < 0.05) were defined as key cells relevant to both stemness and telomere maintenance in BC. Additionally, heterogeneity within these key cells was examined. UMAP-based reclustering was performed using FindNeighbors and FindClusters to delineate cellular subtypes, followed by assessment of biomarker expression across the resulting subclusters (P < 0.05).

### 2.11 Cell communication and pseudo-time analyses of key cells

To infer intercellular interactions, cell–cell communication analysis was conducted by evaluating receptor–ligand expression and pairing patterns among cell types. The distribution and proportions of key cell subtypes relative to other populations in tumor and normal tissues were quantified. Communication networks were constructed using the CellChat package (v1.6.1) (51), generating aggregated intercellular signaling maps and visualizing the contribution of each cell cluster to the overall communication landscape. Developmental trajectories and lineage dynamics of key cells and their subtypes were further explored through pseudo-time analysis using the Monocle package (v2.26.0) (52).

### 2.12 Expression validation of biomarkers

Tumor tissue samples were obtained from five patients diagnosed with BC, alongside five adjacent normal tissue samples from healthy individuals, all collected at The First Affiliated Hospital of Nanjing Medical University. RT-qPCR was performed for experimental validation of biomarker expression. Total RNA was extracted from the ten samples using TRIzol reagent (Ambion, Austin, USA), and cDNA synthesis was carried out using the SureScript First-Strand cDNA Synthesis Kit (Servicebio, Wuhan, China), following manufacturer protocols. qPCR amplification was performed with the 2 × Universal Blue SYBR Green qPCR Master Mix (Servicebio, Wuhan, China). GAPDH served as the internal control, and gene expression was quantified using the 2^−ΔΔCt^ method (53). Primer sequences are provided in **Supplementary Table 1**.

### 2.13 Statistical analysis

All statistical analyses were conducted in R software (v4.2.2). Group comparisons were performed using the Wilcoxon test, with P < 0.05 considered statistically significant.

## 3 Results

### 3.1 Identification of candidate genes and associated pathways

**Figure 2:**
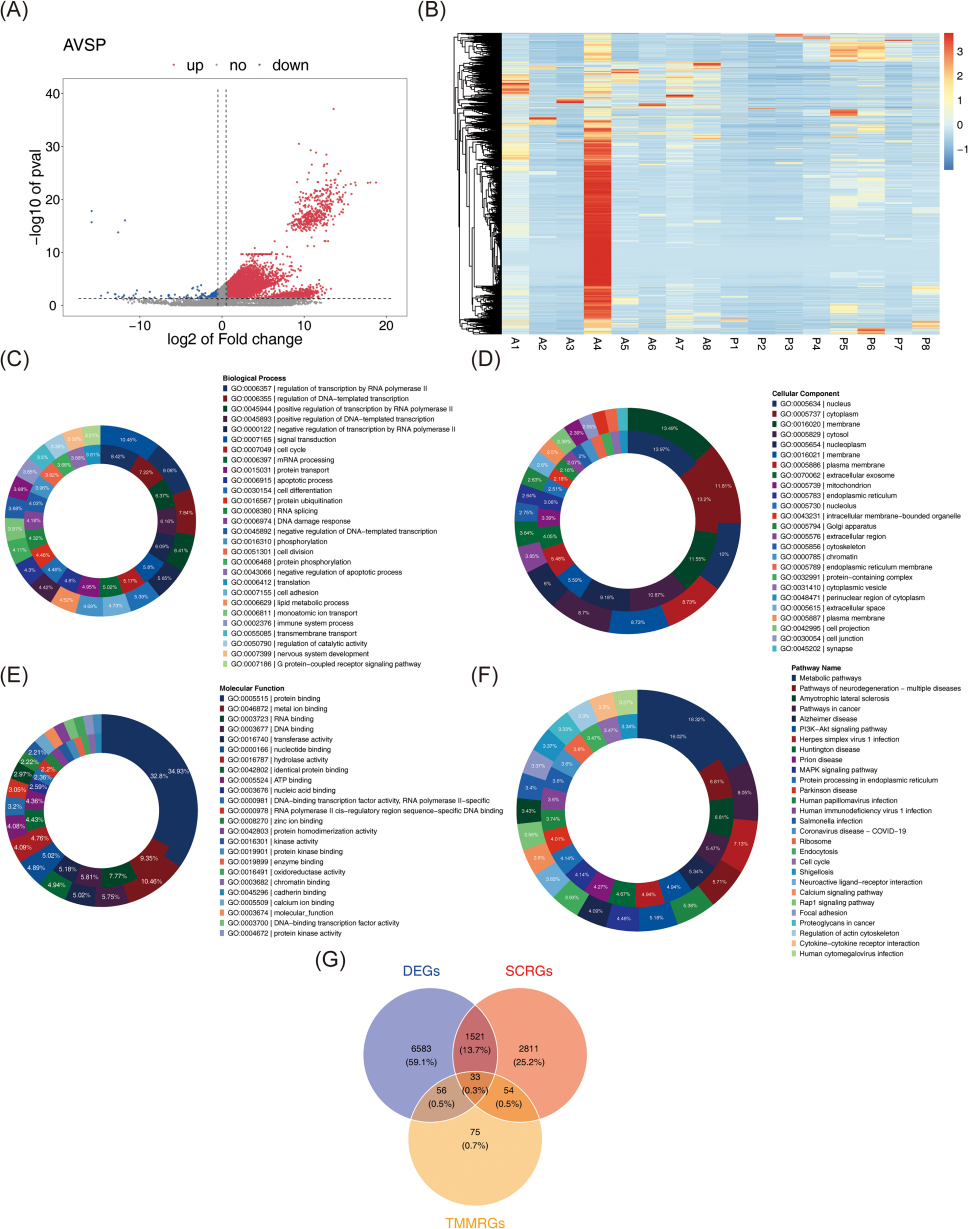
Identification of candidate genes and associated pathways. **(A)** Volcano plots of DEGs between BC samples and normal samples. In the figure, each dot represents a gene. Pink indicates up-regulation of gene expression, while blue indicates down-regulation of gene expression. **(B)** The expression heat map of DEGs. Red represents high expression and blue represents low expression. The darker the color, the higher/lower the expression. **(C-E)** GO analysis with up-regulated (the outer circle) and down-regulated (the inner circle) DEGs, including biological process**(C)**, cellular component **(D)**, and molecular function **(E)**. **(F)** KEGG analysis with up-regulated (the inner circle) and down-regulated (the inner circle) DEGs. **(G)** 33 overlapping genes were identified as both stem cell and telomere maintenance mechanism related DEGs in BC.

Differential expression analysis of the whole transcriptome RNA-seq dataset identified 8,193 DEGs between tumor and normal samples, including 7,014 upregulated and 1,179 downregulated genes in BC (**Figures 2A, B**). These DEGs underwent enrichment analysis, yielding 649 significantly associated GO terms: 404 BPs such as “cell cycle,” “DNA damage response,” and “signal transduction” (**Figure 2C**); 112 CCs, including “plasma membrane,” “extracellular exosome,” and “cell junction” (**Figure 2D**); and 133 MFs, including “protein binding,” “protein kinase activity,” and “DNA-binding transcription factor activity” (**Figure 2E**). Additionally, 68 KEGG pathways enriched in DEGs were identified, including “pathways in cancer,” “PI3K-Akt signaling pathway,” “proteoglycans in cancer,” “MAPK signaling pathway,” and “focal adhesion” (**Figure 2F**). These pathways reflect essential mechanisms contributing to BC pathogenesis, reinforcing the reliability of differential expression results and providing functional insight into molecular alterations associated with BC. By intersecting the 8,193 DEGs with 4,419 SCRGs and 218 TMMRGs, 33 candidate genes related to both stemness and TMM in BC were identified (**Figure 2G**).

### 3.2 Selection of JUN, NFKB1, and SP1 as biomarkers for BC

**Figure 3:**
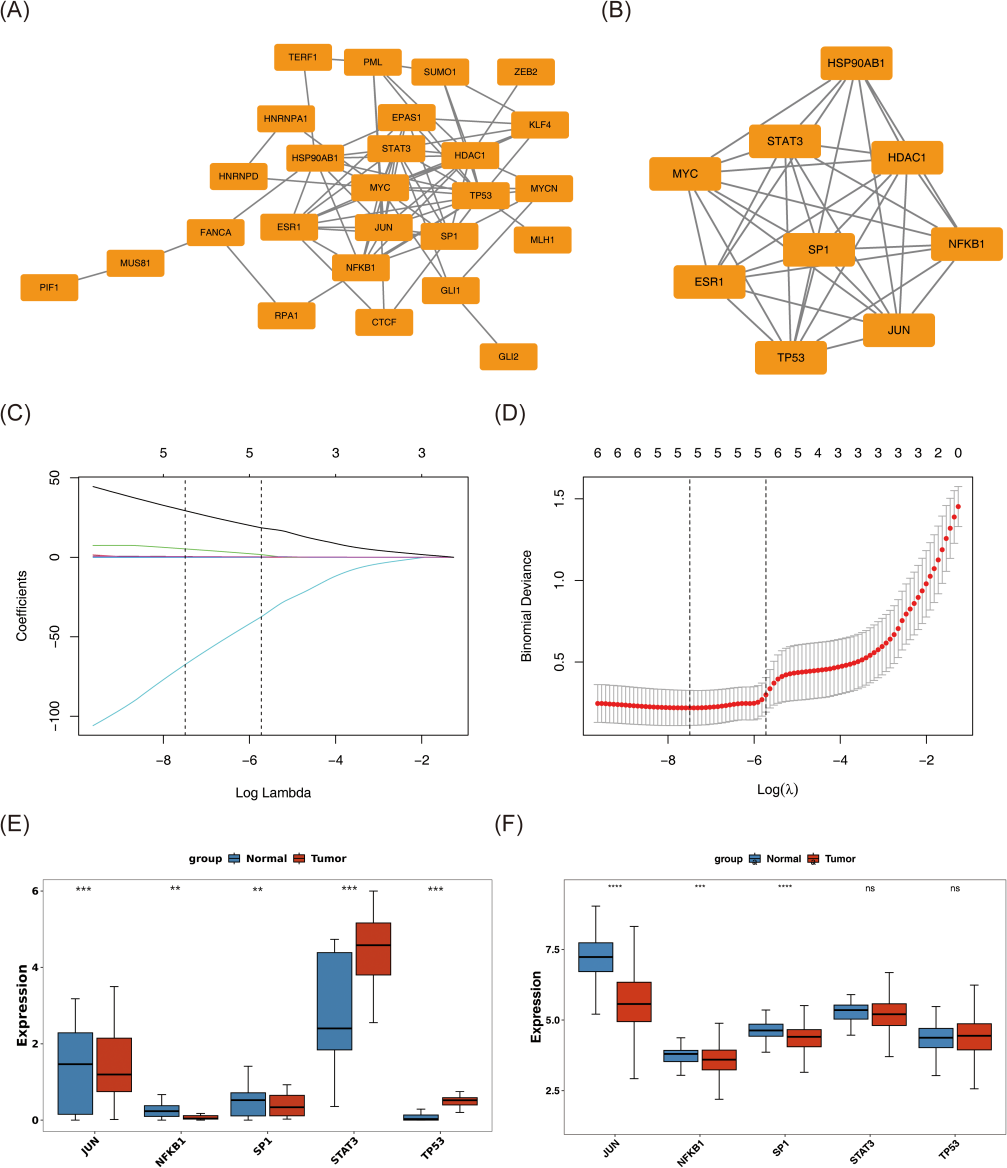
Screening of JUN, NFKB1, and SP1 as biomarkers for BC. **(A)** Protein–protein interaction network of 33 DEGs. **(B)** A significant module containing 9 hub genes was selected using MCODE. **(C, D)** The gene coefficient plot of LASSO analysis and the error plot of cross-validation. The horizontal coordinates were all log(Lambda), and the vertical coordinates were respectively the coefficient of the gene and the error of cross-validation. LASSO algorithm indicates the optimal model contained 5 genes. **(E, F)** The expression levels of these genes in tumor and normal samples were assessed in both whole transcriptome RNA-seq dataset **(E)** and TCGA-BC dataset **(F)** by Wilcoxon rank sum test. The tumor samples were colored red, and the normal samples were colored blue. ns represents P > 0.05, ** represents P < 0.01, *** represents P < 0.001, **** represents P < 0.0001.

A PPI network encompassing 26 nodes and 77 edges was constructed based on these 33 candidate genes using a publicly available database (**Figure 3A**). Further analysis with the MCODE plugin identified the most prominent functional module, comprising 9 hub genes: JUN, HDAC1, HSP90AB1, SP1, ESR1, NFKB1, MYC, STAT3, and TP53. An additional PPI network was generated to visualize the interactions among these hub genes (**Figure 3B**).

Subsequently, the nine hub genes were subjected to LASSO regression analysis. At log(lambda.min) = −7.49352 and log(lambda.1se) = −5.725879, the optimal model retained five genes: JUN, SP1, NFKB1, STAT3, and TP53 (**Figures 3C, D**). Expression profiling across both the whole transcriptome RNA-seq dataset and the TCGA-BC cohort identified JUN, NFKB1, and SP1 as exhibiting statistically significant and consistent expression patterns (P < 0.01) (**Figures 3E, F**). These three genes were subsequently designated as biomarkers associated with both stem cell function and TMM in BC. Notably, all three demonstrated markedly reduced expression in tumor tissues compared to normal counterparts (P < 0.01).

### 3.3 Exploring the pathways by which biomarkers influence BC development

**Figure 4:**
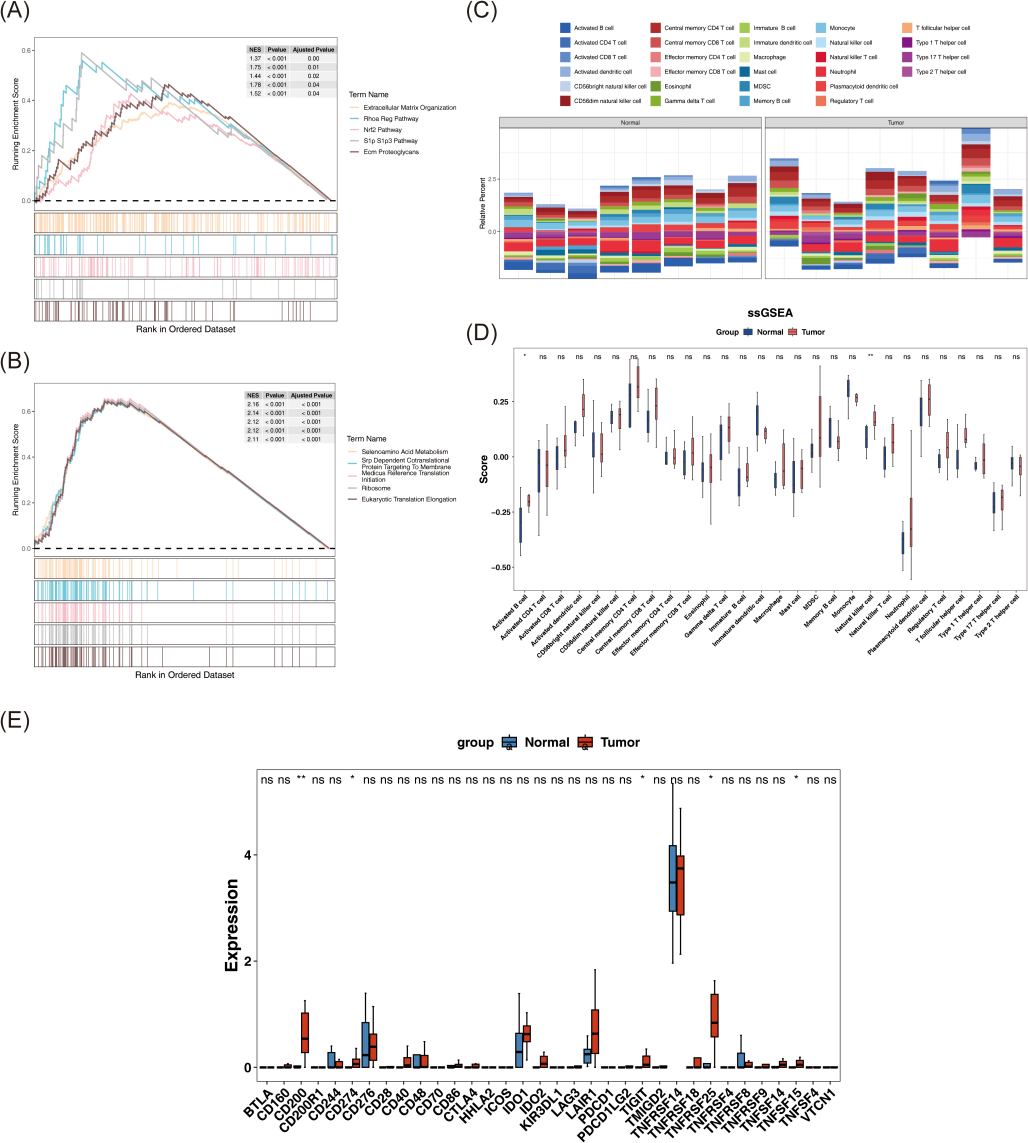
Reveal of signaling pathways associated with biomarkers and potential interaction of immune system in BC patients. **(A, B)** GSEA enrichment results of JUN **(A)** and SP1 **(B)**. Each polyline represents a pathway, and the peak of each polyline is the enrichment fraction of that pathway. **(C)** The relative abundance of 28 immune cells. **(D)** The differences in ssGSEA scores of 28 immune cell types between tumor samples and normal samples were compared by Wilcoxon rank-sum test. **(E)** The expression differences of immune checkpoints between tumor samples and normal samples were compared by Wilcoxon rank-sum test. ns represents P > 0.05, * represents P < 0.05, ** represents P < 0.01.

GSEA was performed to elucidate biological pathways associated with the identified biomarkers. Due to the lack of significant pathway enrichment for NFKB1, downstream analysis focused on pathways enriched by JUN and SP1. Enrichment analysis revealed that JUN was predominantly associated with pathways such as “ECM organization,” “RhoA regulation pathway,” “Nrf2 pathway,” “S1P–S1P3 pathway,” and “ECM proteoglycans” (**Figure 4A**). In contrast, SP1 enriched pathways included “selenoamino acid metabolism,” “SRP-dependent cotranslational protein targeting to membrane,” “translation initiation *via* medicus reference,” “ribosome,” and “eukaryotic translation elongation” (**Figure 4B**). These pathways are involved in key processes including ECM structure and tissue reconstruction, cell signaling regulation, oxidative stress responses, and protein synthesis and transport, all of which may contribute to BC pathogenesis through their interaction with these biomarkers.

### 3.4 Revealing altered immune microenvironments and activated immune checkpoints in patients with BC

Comprehensive analysis of the immune microenvironment in BC revealed distinct immune infiltration patterns, as visualized in a stacked bar chart illustrating the relative abundance of 28 immune cell types across individual samples, computed *via* the ssGSEA algorithm (**Figure 4C**). Notably, activated B cells and NK cells demonstrated statistically significant differences between tumor and normal tissues (P < 0.05), with both cell types showing elevated infiltration in tumor samples (**Figure 4D**). This heightened immune presence likely reflects immune activation within the tumor microenvironment. Specifically, activated B cells may contribute to antibody-mediated responses, while NK cells are known for their direct cytotoxic activity against malignant cells, indicating that differential immune cell infiltration may be associated with immune escape, tumor growth, and the broader pathophysiology of BC.

Expression profiles were available for 34 out of the 40 immune checkpoints identified in the literature. An analysis of these immune checkpoints in tumor and normal samples revealed significant differences in the expression of CD200, CD274, TIGIT, TNFRSF25, and TNFSF15 between the two groups (P < 0.05) (**Figure 4E**). These five immune checkpoints also exhibited elevated expression levels in tumor samples (P < 0.05). This suggests that these checkpoints may be activated within the tumor microenvironment, potentially aiding tumor cells in evading immune surveillance and clearance, thereby positioning them as promising therapeutic targets. Targeting these immune checkpoints could inhibit tumor growth and metastasis.

### 3.5 Investigating targeted drugs and potential molecular mechanisms for biomarkers

**Figure 5:**
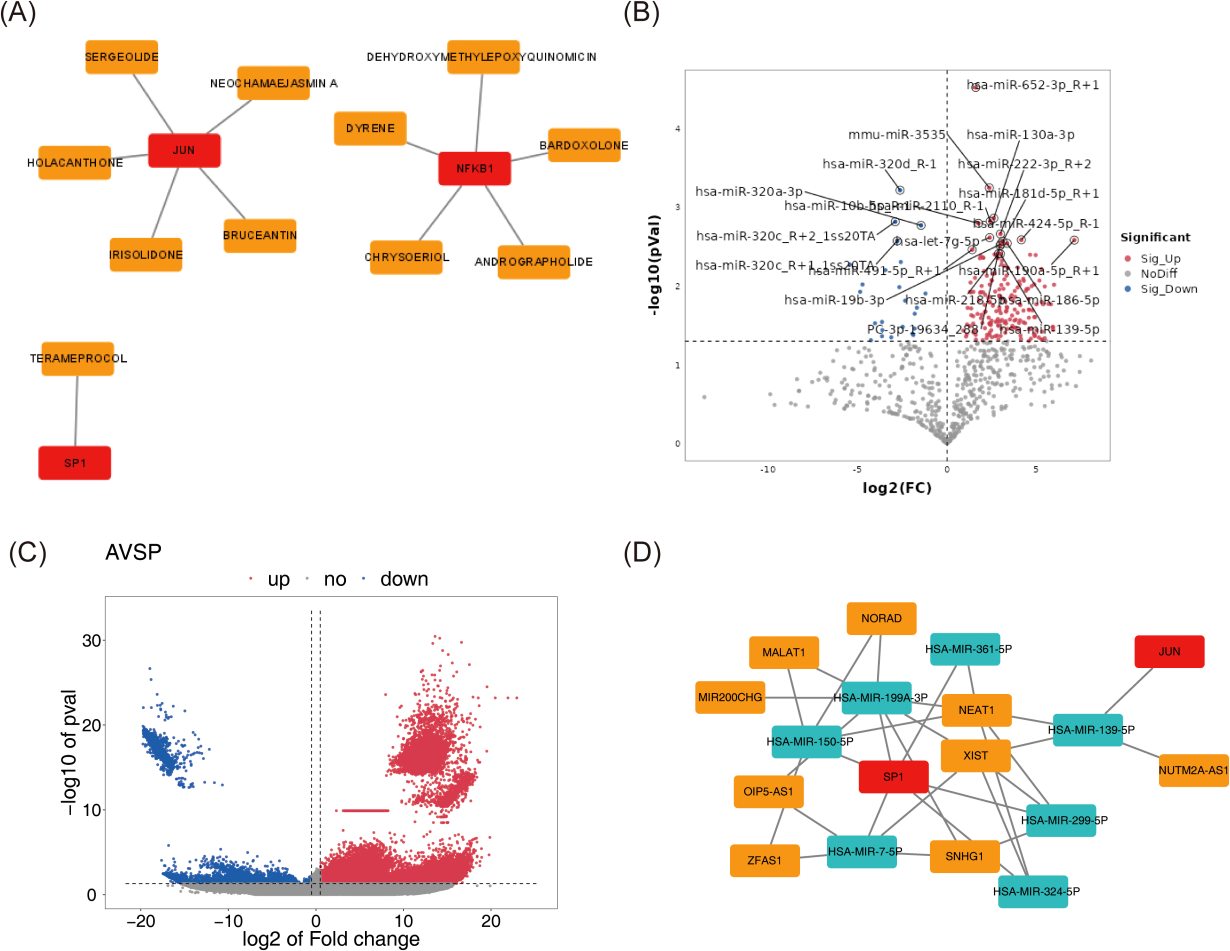
Investigating targeted drugs and potential molecular mechanisms for biomarkers. **(A)** The drug-biomarker network selected by DGIdb. Biomarkers were represented in red, and drugs were represented in orange. **(B, C)** The volcano plot of differentially expressed miRNAs **(B)** and lncRNAs **(C)** between tumor and normal samples. The color pink represented upregulation, while the color blue indicated downregulation. The horizontal axis corresponded to log2FC, denoting the logarithmic value of the gene expression change ratio, and the vertical axis represented the -log10 of the P-value. **(D)** Biomarker-miRNA-lncRNA regulatory network. Red represented biomarkers, cyan indicated miRNAs, and orange denoted lncRNAs.

Utilizing DGIdb, drug–biomarker interactions were predicted and ranked by interaction scores (**Supplementary Table 2**). Based on the selected candidates, a drug–biomarker interaction network was constructed, comprising 14 nodes and 11 edges (**Figure 5A**).

Parallel differential expression analysis identified 272 DE-miRNAs (248 upregulated, 24 downregulated) and 12,287 DE-lncRNAs (11,899 upregulated, 388 downregulated) between tumor and normal samples (**Figures 5B, C**). Predictions of miRNAs targeting the biomarkers, along with their corresponding target lncRNAs, were obtained from public databases. Intersections between DE-miRNAs and predicted target miRNAs, as well as between DE-lncRNAs and predicted target lncRNAs, enabled the identification of key miRNAs and lncRNAs. These regulatory elements were integrated into a lncRNA–miRNA–mRNA network consisting of 18 nodes and 31 edges (**Figure 5D**), including 7 miRNAs, 9 lncRNAs, and 2 biomarkers (SP1 and JUN). Within this network, complex regulatory relationships were delineated; for example, XIST may regulate JUN *via* hsa-miR-139-5p, and potentially modulate SP1 through hsa-miR-199a-3p, hsa-miR-7-5p, hsa-miR-324-5p, and hsa-miR-299-5p.

### 3.6 All 9 annotated cell types exhibited higher counts in BC

**Figure 6:**
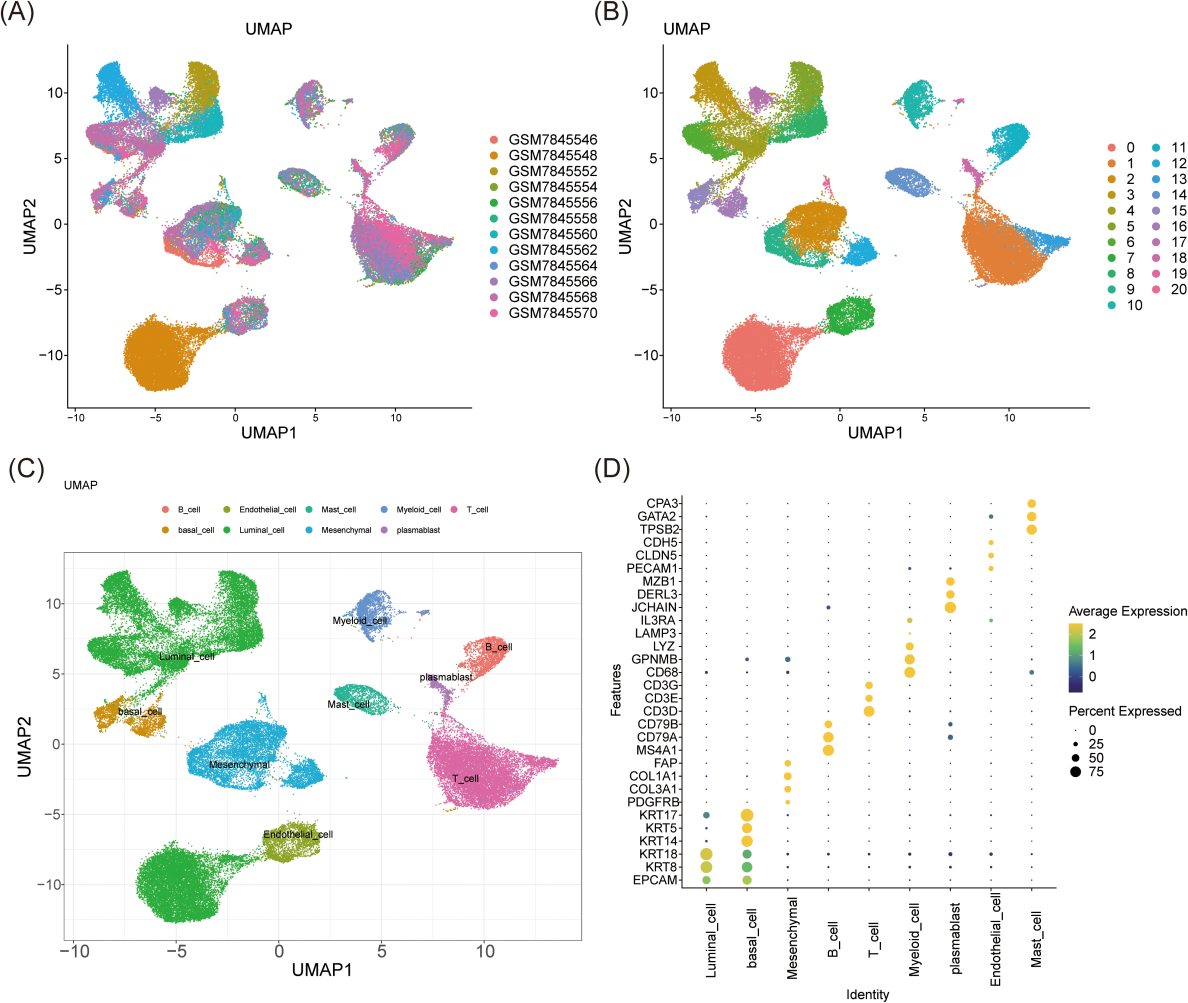
Cell clustering analysis and annotation. **(A, B)** UMAP clustering analysis identified 21 clusters. **(C, D)** The UMAP **(C)** and the bubble plot **(D)** of 9 distinct cell types annotated by marker genes.

Following transcriptomic analysis, single-cell studies provided insights into the unique expression patterns of biomarkers across various cell types and investigated the underlying cellular mechanisms of BC. Data QC was initially performed on the GSE245601 dataset, with metrics for nFeature_RNA, nCount_RNA, and percent.mt visualized before and after QC (**Supplementary Figures 1A, B**). Initially, the dataset contained 69,275 cells and 25,558 genes, which were reduced to 55,204 cells post-filtering, retaining the same number of genes. The top 2,000 highly variable genes were selected for further analysis (**Supplementary Figure 1C**). PCA demonstrated that cells from both tumor and normal samples were concentrated, with minimal variation beyond the 30th principal component, leading to the selection of the first 30 components (dims = 30) and a clustering resolution of 0.4 (**Supplementary Figures 1D, E**). UMAP clustering revealed 21 distinct clusters (**Figures 6A, B**). Marker gene analysis identified nine cell types: plasmablasts, mast cells, B cells, myeloid cells, basal cells, endothelial cells, mesenchymal cells, T cells, and luminal cells (**Figure 6C**). The specificity of these marker genes was validated using a bubble plot, enabling precise annotation of cell types (**Figure 6D**). The distribution of these nine cell types in tumor and normal samples was also visualized, revealing similar clustering patterns between groups but with higher cell counts in tumors across all types compared to normal samples (**Supplementary Figure 2**). These differences are likely attributed to the tumor microenvironment and the activation of immune responses in patients with BC. Additionally, basal cells had higher scores in the ERK-JUN signaling and stemness-related signatures pathways, while myeloid cells had the highest score in the NF-κB activation pathway (**Supplementary Figure 3**). This suggested that the proliferation, differentiation, and stemness regulation of basal cells, as well as the inflammatory response of myeloid cells, might play important roles in the biological processes of BC.

### 3.7 Defining mesenchymal cells as a key cell population

**Figure 7:**
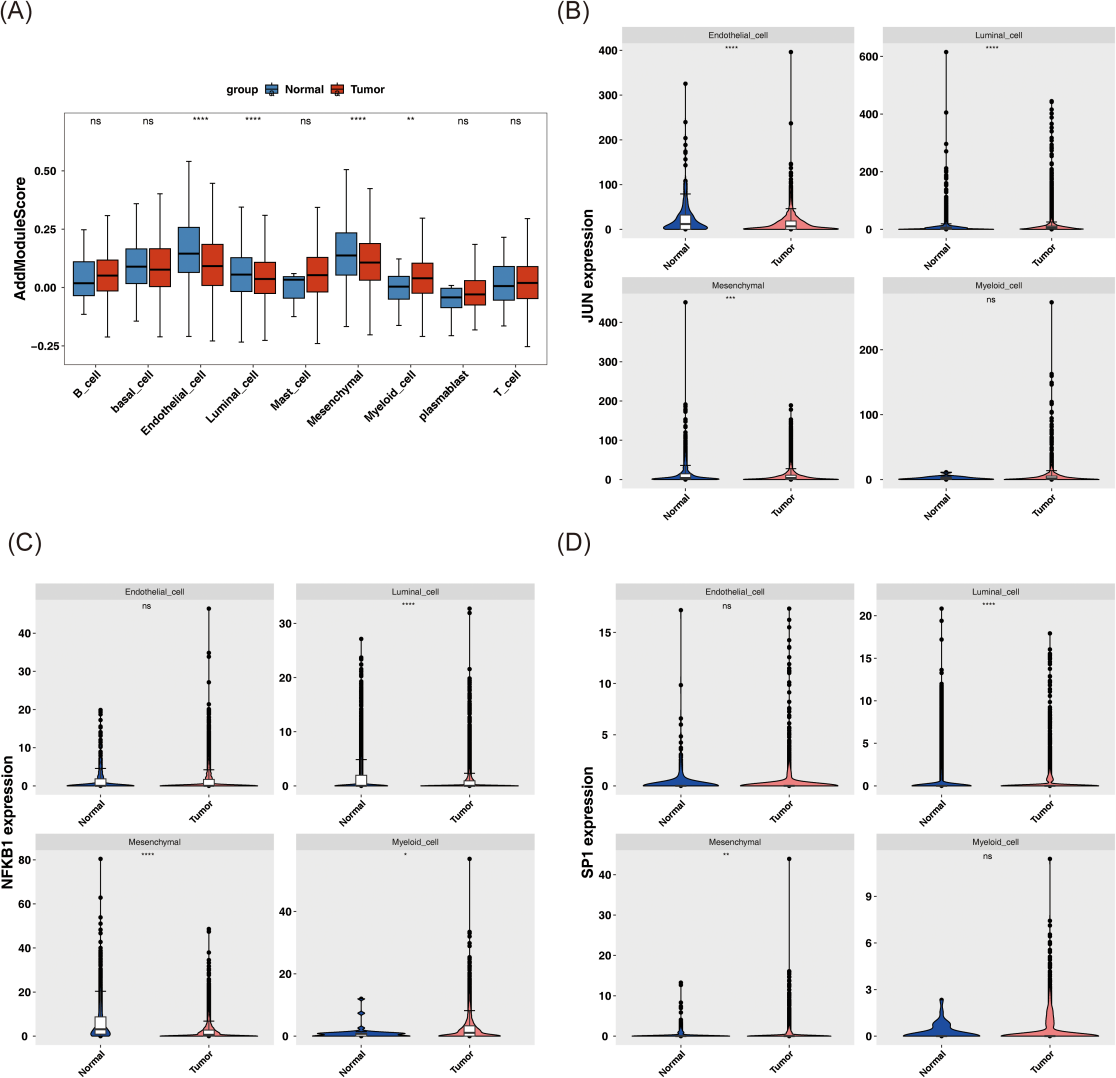
Analysis of key cell biomarkers. **(A)** AddModuleScore score of different cell types in tumor and normal samples. **(B-D)** The expression patterns of JUN **(B)**, NFKB1 **(C)**, and SP1 **(D)** in these candidate key cells in tumor and normal samples. ns represents P > 0.05, * represents P < 0.05, ** represents P < 0.01, *** represents P < 0.001, **** represents P < 0.0001.

Subsequent analyses employed the AddModuleScore function, using the previously identified 33 candidate genes associated with stem cells and TMM in BC as the background gene set. The scores for the nine annotated cell types in tumor and normal samples were calculated and compared. Noteworthy differences in AddModuleScore were observed among endothelial cells, luminal cells, mesenchymal cells, and myeloid cells (P < 0.01), indicating alterations in cell function and state within the tumor microenvironment. These cells were thus classified as candidate key cells (**Figure 7A**). Specifically, endothelial, luminal, and mesenchymal cells exhibited significantly lower scores in tumor samples (P < 0.0001), suggesting suppression of their stem cell properties and TMM within the tumor microenvironment. This suppression may be linked to tumor aggressiveness, metastatic potential, or treatment resistance. In contrast, myeloid cells displayed significantly higher scores in tumor samples (P < 0.01), potentially reflecting their active role in supporting tumor growth and maintaining the tumor microenvironment, such as by promoting inflammation, enhancing tumor cell survival, or suppressing immune responses.

Further investigation of the expression patterns of JUN, NFKB1, and SP1 in these key cell types revealed significant differences in both mesenchymal and luminal cells (P < 0.01) (**Figures 7B–D**).

### 3.8 Recognition of mesenchymal cell subtypes: MSC1, MSC2, and MSC3

**Figure 8:**
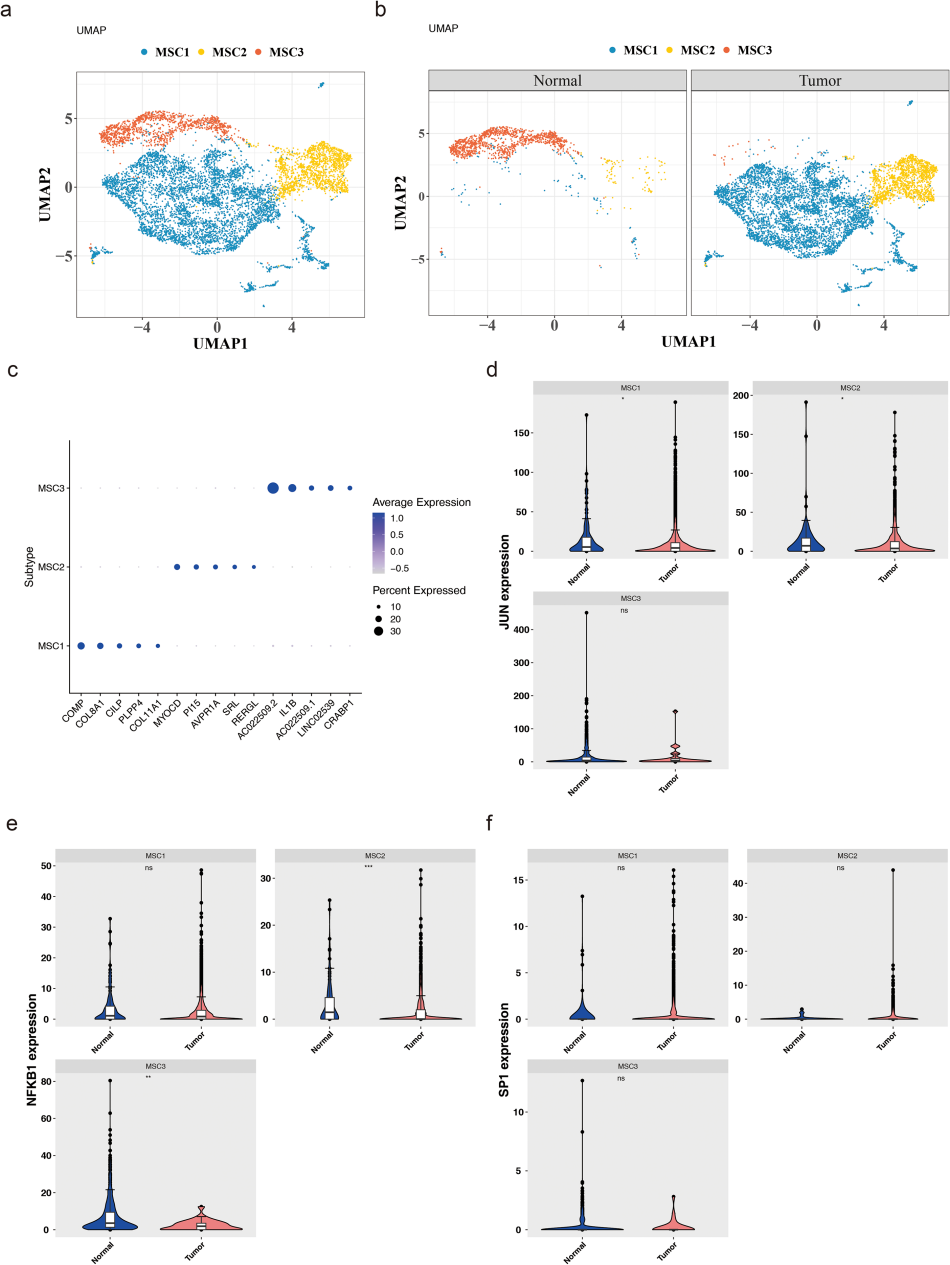
Heterogeneity of mesenchymal cells. **(a)** Mesenchymal cells can be further divided into three cell subtypes. **(b)** The expression of these subtypes in tumor and normal tissues. **(c)** The specificity of marker genes across these 3 subtypes. **(d-f)** The expression levels of JUN **(d)**, NFKB1 **(e)** and SP1 **(f)** in these subtypes. *:p<0.05; **:p<0.01; ***:p<0.001; ns:p>0.05

Subsequent investigations examined the heterogeneity of mesenchymal cells, with UMAP clustering categorizing these cells into three subtypes: MSC1, MSC2, and MSC3 (**Figure 8a**). The distribution of these subtypes in tumor and normal tissues revealed that, compared to normal samples, MSC1 and MSC2 were more abundant in BC, while MSC3 was less prevalent (**Figure 8b**). A bubble plot further emphasized the specificity of marker genes across the three subtypes (**Figure 8c**). Biomarker expression was then evaluated across these subtypes. In tumor samples, JUN exhibited significantly lower expression in MSC1 and MSC2 (P < 0.05), while NFKB1 was significantly reduced in MSC2 and MSC3 (P < 0.01) (**Figures 8d, e**). SP1, however, showed no significant expression differences across the three subtypes (P > 0.05) (**Figure 8f**).

### 3.9 Analyzing communication relationships between mesenchymal cell subtypes and other cell types

**Figure 9:**
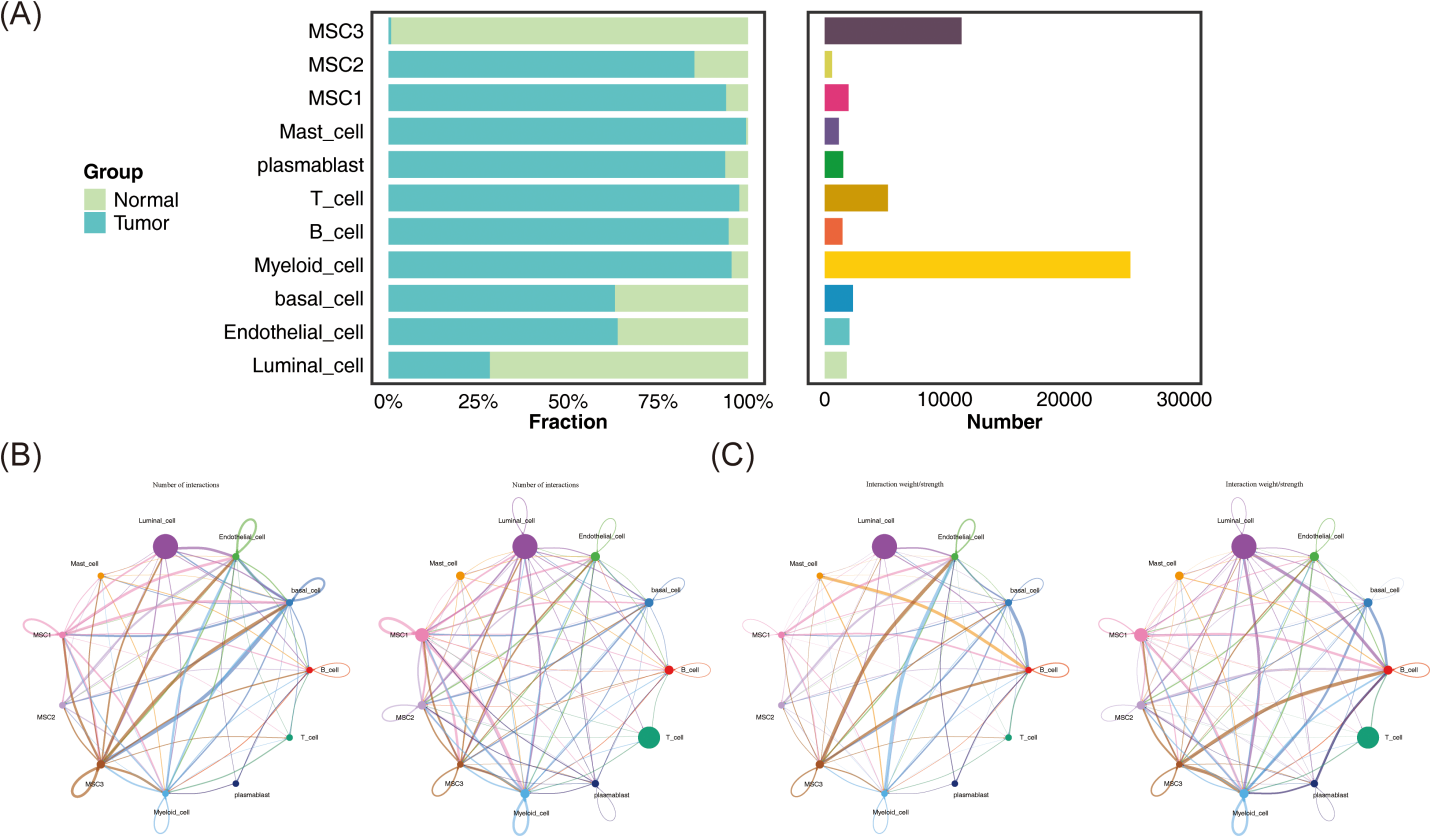
Communication relationships between mesenchymal cell subtypes and other cell types. **(A)** The distribution and quantity of different cell types in tumor and normal tissues. **(B)** The interaction numbers between these cell types in tumor and normal tissues. **(C)** The weight of interactions between these cell types in tumor and normal tissues.

The study further assessed the distribution and abundance of three mesenchymal cell subtypes, alongside eight other cell types, in both tumor and normal tissues, revealing significant variations in cell type proportions between these environments (**Figure 9A**). These differences suggested that BC may influence the interaction dynamics among various cell populations. Subsequently, communication analysis was conducted to evaluate interactions between MSC1, MSC2, and MSC3, as well as the other eight cell types, under both normal and tumor conditions, focusing on both the frequency and strength of these interactions. Under BC conditions, the number of interactions between the mesenchymal subtypes and other cell types was generally reduced compared to normal tissues (**Figure 9B**), implying that the tumor microenvironment may alter cell functionality and behavior by diminishing communication between mesenchymal cells and other cell types.

**Figure 10:**
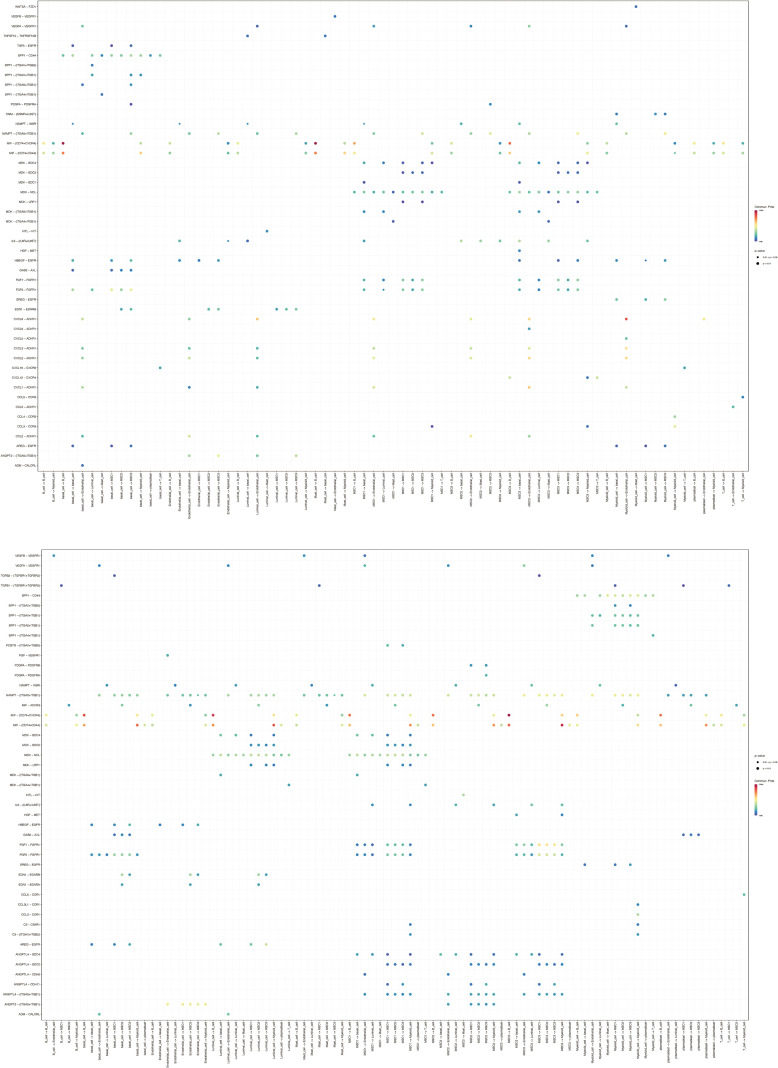
Ligand-receptor interactions among these cell types under control and BC conditions.

In terms of interaction strength, compared to normal conditions, communication between MSC1 and endothelial cells was weakened in BC, while interactions with B cells and myeloid cells were enhanced. Similarly, MSC2 showed reduced interaction with endothelial cells but increased communication with basal and myeloid cells. MSC3 exhibited similar patterns, with decreased communication with endothelial cells and increased interaction with myeloid cells (**Figure 9C**). These findings may reflect alterations in immune regulation within the tumor microenvironment, potentially contributing to tumor invasiveness and environmental changes. The results suggest that BC could reorganize functional and interaction patterns among cells, particularly between mesenchymal and immune cells, which may be critical for understanding tumor progression and developing effective therapeutic strategies. Additionally, ligand-receptor interactions among the mesenchymal cell subtypes and other cell types under both normal and BC conditions were also examined (**Figure 10**), highlighting the importance of these interactions in regulating cellular behavior, maintaining tissue function, and responding to environmental changes.

### 3.10 Pseudo-time trajectory inference of mesenchymal cells

**Figure 11:**
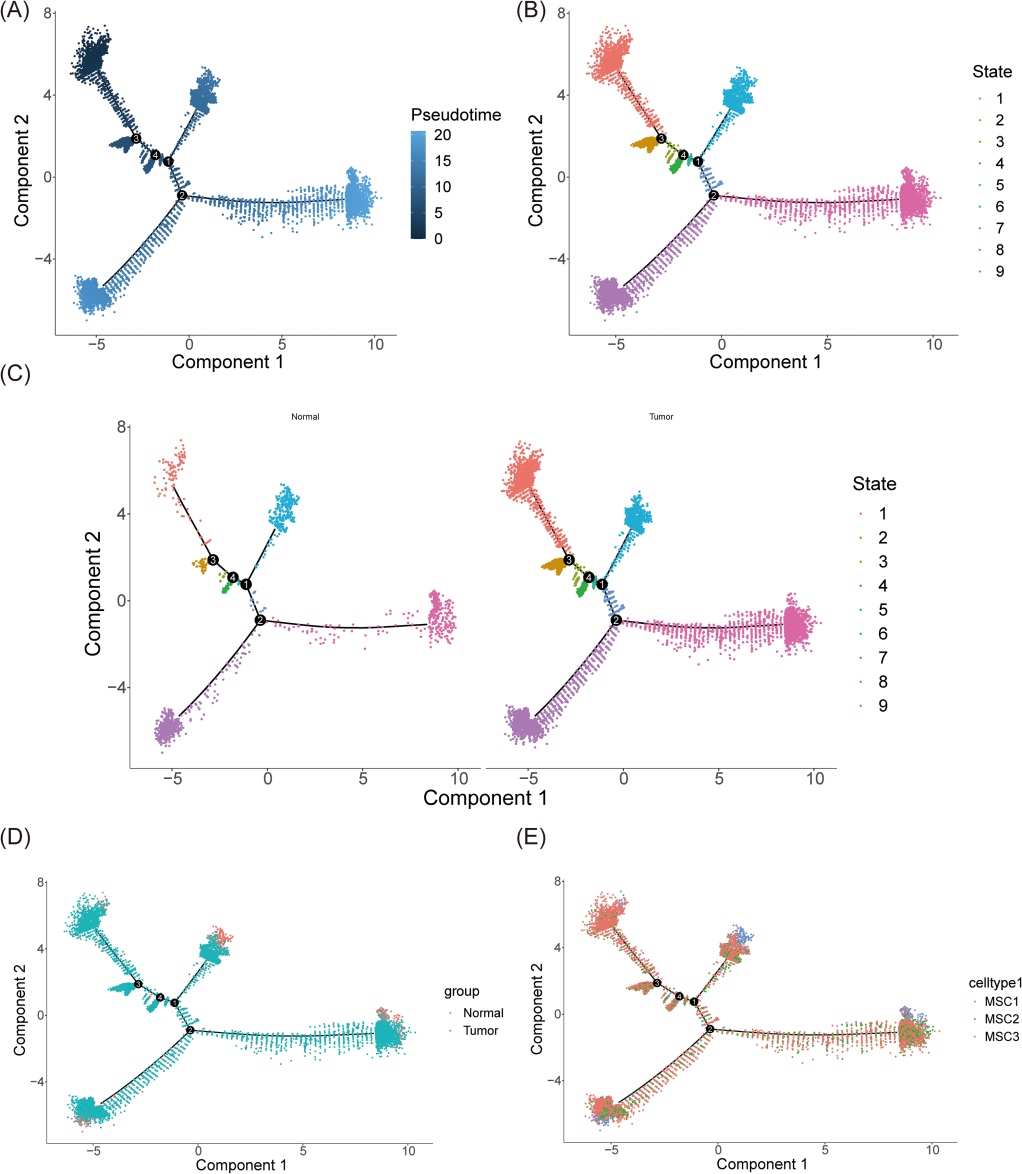
Pseudo-time trajectory inference of mesenchymal cells. **(A)** The trajectory plot illustrated the differentiation of mesenchymal cells over time. During the differentiation process, mesenchymal cells were divided into four distinct stages. Darker colors represented earlier time points in the differentiation timeline. **(B)** The trajectory was categorized into 9 stages. **(C, D)** The trajectory of mesenchymal cells in normal and tumor tissues respectively **(C)** and synthetically **(D)**. **(E)** The distribution of 3 mesenchymal cell subtypes along the differentiation trajectory.

In the final stages of the study, pseudo-time analysis was performed on mesenchymal cells to trace their developmental trajectory. The analysis identified a clear starting point, from which cells underwent maturation as they progressed along the trajectory. Four differentiation nodes were detected, signaling points where individual cell clusters began to diverge into distinct cellular states or fates, underscoring the heterogeneity within mesenchymal cells that guides their progression through various developmental paths (**Figure 11A**). The mesenchymal cell trajectory was divided into nine stages, with cells evenly distributed across early, middle, and late developmental stages, highlighting the continuous and complex nature of mesenchymal cell development (**Figure 11B**).

Notably, under BC conditions, the number of mesenchymal cells across all developmental stages significantly increased, corroborating previous findings (**Figures 11C, D**). This suggests that the BC microenvironment promotes an expansion of mesenchymal cells, enhancing their differentiation potential and possibly contributing to tumor progression and the remodeling of the tumor microenvironment. The study also depicted the distribution of the three mesenchymal subtypes—MSC1, MSC2, and MSC3—along the mesenchymal cell differentiation trajectory (**Figure 11E**). These subtypes were present throughout all stages, indicating the intrinsic heterogeneity and complex interactions among mesenchymal cells within the tissue. Interestingly, MSC1 was predominantly more abundant at every stage, possibly reflecting its central role in mesenchymal cell development and function. This suggests that MSC1 may possess greater biological activity or functionality, influencing both neighboring cells and the broader cellular community.

### 3.11 Verification of biomarkers expression

**Figure 12:**
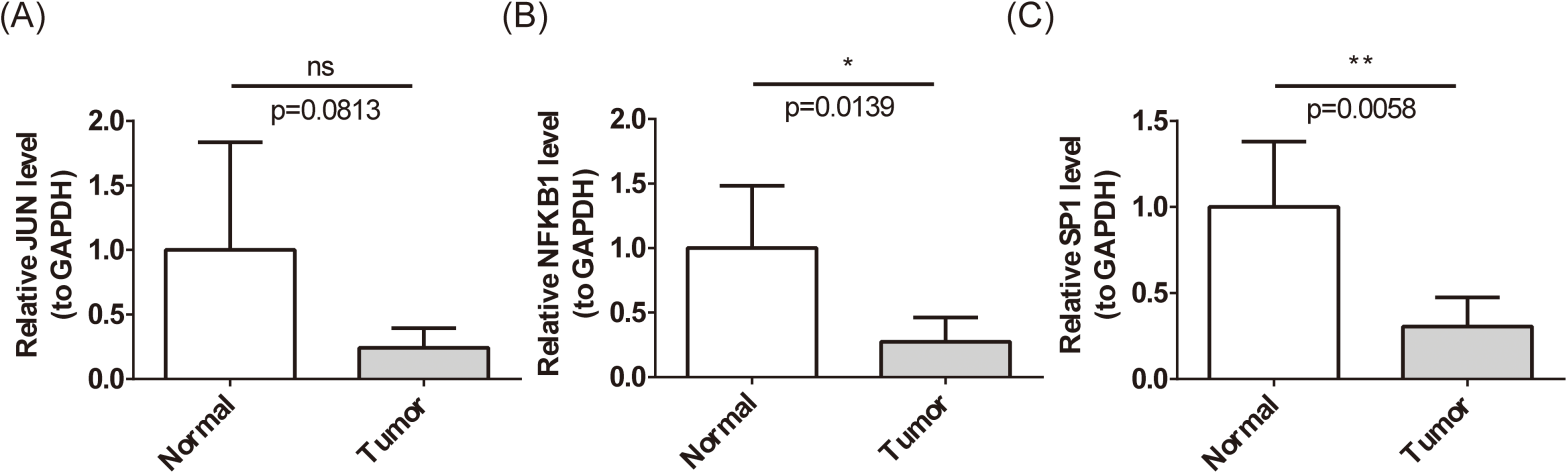
Expression level of biomarkers’ mRNA. **(A-C)** Expression levels of JUN **(A)**, NFKB1 **(B)**, and SP1 **(C)** in patients with BC by RT-qPCR. ns represents P > 0.05, * represents P < 0.05, ** represents P < 0.01. ns means 'not significant', and it represents p>0.05.

Previous analyses revealed significantly lower expression levels of JUN, NFKB1, and SP1 in BC samples from both the whole transcriptome RNA-seq dataset and the TCGA-BC dataset (P < 0.01) (**Figures 3E, F**). To further validate these findings at the clinical level, RT-qPCR was employed to assess the expression of these biomarkers in patients with BC (**Figures 12A–C**). RT-qPCR results confirmed that both NFKB1 and SP1 exhibited significantly reduced expression in BC samples (P < 0.05), aligning with initial observations. Although JUN demonstrated a trend toward decreased expression in BC, the difference was not statistically significant (P = 0.0139).

## 4 Discussion

BC is a multifactorial disease, influenced by various biological factors, with SCRGs and TMMRGs playing critical roles in tumor progression through the enhancement of tumor cell proliferation and self-renewal capabilities (23). Understanding the interactions between these mechanisms is crucial for identifying potential therapeutic targets and improving patient outcomes. This study highlights JUN, NFKB1, and SP1 as biomarkers with significantly reduced expression levels in BC tissues. These biomarkers are implicated in the progression of BC through various biological processes, such as ECM remodeling, cell signaling regulation, oxidative stress response, and mechanisms governing protein synthesis and transport. Notably, scRNA-seq analysis revealed high infiltration levels of activated B cells and NK cells in BC, alongside elevated expression of immune checkpoints, including CD200, CD274, TIGIT, TNFRSF25, and TNFSF15.

JUN promotes the self-renewal and stemness maintenance of cancer stem cells by activating stem cell-related pathways such as β-catenin and Notch and enhances tumor initiation ability and chemotherapy resistance (54, 55). Abnormal activation of telomerase reverse transcriptase (TERT) in cancer cells enables them to escape cellular senescence and thereby acquire unlimited proliferation ability. JUN may indirectly promote the expression of TERT, activate telomerase activity, compensate for telomere shortening during cell division, maintain chromosomal stability, and assist in the unlimited proliferation of cancer cells (56, 57). NFKB1 encoded a key component of the NF-κB transcription factor complex, which was critical in the occurrence and development of various diseases including cancer (58). Moreover, NFKB1 is involved in regulating the self-renewal, differentiation and treatment resistance of cancer stem cells (59). In addition, studies have shown that NFKB1 has a subunit specific role in the DNA damage response (60). This suggests that NFKB1 may indirectly affect the stability of telomeres by influencing the DNA damage repair pathway. SP1 has also been found to be related to the characteristics of cancer stem cells. For example, in triple-negative breast cancer (TNBC), SGCE promotes BC stemness by facilitating the transcription of FGF-BP1 by SP1 (61). Furthermore, Liu et al. found that SP1 mediated the inhibition of TERT after the overexpression of LKB1, thereby affecting the progression of lung adenocarcinoma. That is to say, SP1 might also affect TERT through a similar mechanism in BC and thereby influence the maintenance mechanism of telomeres. In conclusion, JUN, NFKB1 and SP1 may jointly maintain the characteristics of cancer stem cells by regulating the stemness maintenance and telomerase activity of cancer stem cells, thus influencing the progression, treatment resistance and recurrence of BC.

Moreover, JUN, SP1 and NFKB1 play multiple roles in the regulation of the immune microenvironment of BC. JUN, as a kernel factor of the AP-1 family, promotes the invasion of tumor cells by activating Matrix Metalloproteinases and at the same time down-regulates the expression of MHC-I class molecules to evade the recognition of cytotoxic T cells; the secretion of IL-6/IL-8 induced by it can also recruit immunosuppressive myeloid cells and inhibit T cell function (62–64). SP1 regulates the GC-rich region of the PD-L1 promoter, enhances its transcriptional level, and mediates tumor immune escape (65, 66). Meanwhile, it can promote the expression of chemokine CCL2, recruit immunosuppressive macrophages, and affect the immune microenvironment (67). NFKB1 can continuously activate the NF-κB pathway. The CXCL12/CXCR4 axis NFBK1 regulates can also promote the infiltration of regulatory T cells (Tregs) and inhibit anti-tumor immunity (68–70). They three jointly reshape the immune microenvironment through the transcriptional regulatory network and weaken the body’s anti-tumor immune response, which may become a potential mechanism for drug resistance to immunotherapy in breast cancer.

Immune infiltration analysis revealed a significant increase in the abundance of activated B cells and NK cells in BC tumor samples compared to normal tissue (P < 0.05). This elevated infiltration suggests that the immune system is activated within the BC microenvironment, potentially reflecting the body’s attempt to combat tumor progression. B cells are important components of adaptive immunity and can mediate the tumor cells killing by generating antibodies (71). B cells can also act as antigen-presenting cells (APC), presenting tumor antigens to T cells and activating the anti-tumor immune response of T cells (72). Multiple studies have shown that B-cell infiltration is associated with a favorable prognosis in cancer patients (73, 74). A higher level of activated B cell infiltration may indicate a stronger anti-tumor immune response and better clinical outcomes. NK cells are important components of the innate immune system, capable of recognizing and killing tumor cells without prior sensitization (75, 76). Moreover, studies have shown that activated NK cells may play a significant role in early tumor control (77), thereby improving the survival rate of patients. In conclusion, B cells and NK cells exert immune effects respectively by mediating antibody killing, antigen presentation and direct killing of tumor cells. Their activation degree and the infiltration level are significantly associated with the prognosis of cancer patients.

Furthermore, significant differences in the expression of immune checkpoints—specifically CD200, CD274 (PD-L1), TIGIT, TNFRSF25, and TNFSF15—were observed between tumor and normal samples. All five immune checkpoints showed elevated expression in tumor samples (P < 0.05), suggesting their activation within the tumor microenvironment, which likely aids tumor cells in evading immune surveillance. CD200 functions as an immunosuppressive molecule through its receptor CD200R, and its upregulation in BC may facilitate immune escape and contribute to tumor progression and metastasis. PD-L1, a key immune checkpoint, interacts with PD-1 on T cells when expressed on tumor cells, leading to T cell inhibition. High PD-L1 levels in BC are associated with poor prognosis, and blocking PD-L1/PD-1 interactions has emerged as an effective treatment for various BC subtypes. The elevated expression of these immune checkpoints highlights their potential as therapeutic targets in BC. Inhibiting these checkpoints could restore immune surveillance and enhance the efficacy of immune-based therapies (78). This immune analysis highlights the complex interplay between immune cells and the tumor microenvironment in BC. The increased infiltration of activated B cells and NK cells suggests an active immune response, while the upregulation of specific immune checkpoints points to mechanisms of immune evasion. Understanding these dynamics is essential for developing novel immunotherapeutic strategies aimed at boosting anti-tumor immunity while counteracting immune suppression. Future research should focus on elucidating the roles of these immune components and their interactions in BC, as well as exploring combination therapies that could synergistically improve treatment outcomes.

This study explored the lncRNA-miRNA-mRNA regulatory network in BC, revealing interactions between non-coding RNAs and key biomarkers (79, 80). For instance, XIST was predicted to regulate JUN through hsa-miR-139-5p, and may also influence SP1 *via* several miRNAs, including hsa-miR-199a-3p, hsa-miR-7-5p, hsa-miR-324-5p, and hsa-miR-299-5p. These interactions suggest complex regulatory mechanisms in which non-coding RNAs play pivotal roles in modulating the expression of these biomarkers. Given that this network was constructed based on differential expression analysis and database predictions, the reliability of these regulatory relationships is notably high. Previous research has demonstrated that the loss of OPA interacting protein 5 can inhibit BC proliferation *via* the hsa-miR-139-5p/NOTCH1 axis (81), and hsa-miR-7-5p has also been implicated in BC progression (82). Our findings further support the potential of targeting these non-coding RNA interactions for therapeutic intervention in BC. Specifically, the results suggest that XIST and the identified miRNAs (e.g., hsa-miR-139-5p and hsa-miR-7-5p) could jointly regulate SP1 and JUN, thereby influencing key cellular processes such as proliferation, apoptosis, and metastasis, which contribute to BC development. This highlights the potential co-regulatory roles of non-coding RNAs within the tumor microenvironment and their importance in BC progression.

Additionally, the study identified several drug candidates targeting these biomarkers, with Bardoxolone (83), Andrographolide (84), and Terameprocol (85, 86) emerging as potentially effective agents in BC treatment. These drugs target critical pathways involved in oxidative stress, apoptosis, and transcriptional regulation, positioning them as promising candidates for further investigation. However, additional *in vivo* and *in vitro* studies, along with large-scale clinical trials, are necessary to validate their efficacy and safety in BC.

This study further identified three mesenchymal cell subtypes—MSC1, MSC2, and MSC3—each displaying distinct expression patterns of BC biomarkers. JUN was significantly downregulated in MSC1 and MSC2, while NFKB1 was downregulated in MSC2 and MSC3. However, SP1 did not show significant expression differences across these subtypes. The downregulation of JUN and NFKB1 in mesenchymal subtypes may impair their ability to maintain tumor-suppressive functions, highlighting their potential role in driving BC progression. In addition, studies have shown that the MSC1 subtype may have pro-inflammatory properties and play a specific role in immune regulation (87). For example, MSCS activated by Toll-like receptor 4 ligand lipopolysaccharide may present the MSC1 phenotype and express pro-inflammatory cytokines such as IL-6 and IL-8 (87, 88). The MSC2 subtype may have immunosuppressive and tissue repair functions (87, 89). It is mainly produced in an anti-inflammatory environment and is characterized by high expression of immunosuppressive molecules such as IDO, PGE2 and TGF-β, which can inhibit the activity of immune cells and alleviate the inflammatory response (87, 89, 90). However, there are relatively few studies on the MSC3 subtype, and its specific functions remain to be further clarified. The heterogeneity of these mesenchymal subtypes was further explored through communication and pseudo-time analysis. Notably, MSC1 and MSC2 were more abundant in tumor tissues, suggesting that these subtypes may play an active role in tumor progression. The reduced interactions between these mesenchymal subtypes, along with enhanced communication with immune cells such as B cells and myeloid cells under BC conditions, suggest that the tumor microenvironment alters mesenchymal cell functionality. These changes may contribute to immune suppression, tumor cell survival, and sustained tumor growth (91, 92).

Pseudo-time analysis also revealed that mesenchymal cells undergo continuous differentiation across nine distinct stages, emphasizing their developmental plasticity. This trajectory analysis indicated that BC significantly increases the number of mesenchymal cells at all stages of development, further underscoring their critical role in tumor development. MSC1, in particular, was the most prominent subtype across all stages, potentially due to its central role in mesenchymal cell function and its influence on surrounding cell types within the tumor microenvironment (92, 93). Overall, mesenchymal cells appear to contribute to several aspects of BC pathogenesis, including the establishment of the tumor microenvironment, invasion, metastasis, and resistance to treatment. Their ability to interact with immune cells and modulate the ECM suggests that targeting these cells could disrupt cancer-supportive processes. Additionally, targeting epithelial-mesenchymal transition (EMT) and mesenchymal-epithelial transition (MET) pathways in mesenchymal cells may offer new therapeutic strategies to inhibit tumor metastasis and progression in BC.

In summary, this study identified JUN, NFKB1, and SP1 as important biomarkers associated with stem cells and telomere maintenance mechanisms in BC, highlighting their critical roles in tumor progression, alterations in the immune microenvironment, and potential therapeutic targets. Additionally, the exploration of mesenchymal cell heterogeneity and their dynamic communication with other cell types provides valuable insights into the complexity of the tumor microenvironment. However, the study has certain limitations, particularly the small sample size used for validation, which may restrict the generalizability of the results. To address this, we will actively expand the sample sources, collaborate with multiple centers, and incorporate more clinical samples for analysis and validation in the future. Meanwhile, future research should focus on integrating bioinformatic predictions with experimental validation to establish direct evidence for the involvement of JUN, SP1, and NFKB1 in telomere maintenance and stemness regulation.

## 5 Conclusions

In conclusion, this study provides valuable insights into the roles of mesenchymal and luminal cells in BC, while also underscoring the need for further research to validate and expand these findings. Addressing the limitations of the current study will be critical for enhancing our understanding of the underlying mechanisms and improving treatment outcomes for patients with BC.

## Data Availability

The datasets presented in this study can be found in online repositories. The names of the repository/repositories and accession number(s) can be found in the article/**Supplementary Material**.

## Glossary

SCRGs: Stem Cell-Related Genes
TMMRGs: Telomere Maintenance Mechanism-Related Genes
BC: Breast Cancer
RNA-seq: RNA Sequencing
scRNA-seq: Single-Cell RNA Sequencing
DEGs: Differentially Expressed Genes
PPI: Protein-Protein Interaction
MCODE: Molecular Complex Detection
LASSO: Least Absolute Shrinkage and Selection Operator
GO: Gene Ontology
KEGG: Kyoto Encyclopedia of Genes and Genomes
GSEA: Gene Set Enrichment Analysis
ssGSEA: Single-Sample Gene Set Enrichment Analysis
TCGA: The Cancer Genome Atlas
GEO: Gene Expression Omnibus
RT-qPCR: Reverse Transcription Quantitative Polymerase Chain Reaction
ECM: Extracellular Matrix
NK cells: Natural Killer cells
MSC: Mesenchymal Stem Cells
EMT: Epithelial-Mesenchymal Transition
MET: Mesenchymal-Epithelial Transition
PCA: Principal Component Analysis
UMAP: Uniform Manifold Approximation and Projection
FPKM: Fragments Per Kilobase of transcript per Million mapped reads
BP: Biological Process
CC: Cellular Component
MF: Molecular Function
lncRNA: Long Non-Coding RNA
miRNA: MicroRNA
mRNA: Messenger RNA
DGIdb: Drug Gene Interaction Database
TLS: Tertiary Lymphoid Structures
IFN: Interferon
TNF: Tumor Necrosis Factor
PD-L1: Programmed Death-Ligand 1
PD-1: Programmed Cell Death Protein 1
TIGIT: T cell Immunoreceptor with Ig and ITIM domains
TNFRSF25: Tumor Necrosis Factor Receptor Superfamily Member 25
TNFSF15: Tumor Necrosis Factor Superfamily Member 15
CD200: Cluster of Differentiation 200
CD274: Cluster of Differentiation 274
Nrf2: Nuclear factor erythroid 2–related factor 2
RhoA: Ras homolog family member A
S1P: Sphingosine-1-phosphate
SRP: Signal Recognition Particle
NF-κB: Nuclear Factor kappa-light-chain-enhancer of activated B cells
STAT3: Signal Transducer and Activator of Transcription 3
TP53: Tumor Protein p53
TMM: Telomere maintenance mechanisms
TERT: Telomerase reverse transcriptase
TNBC: Triple-negative breast cancer
MDSCs: Myeloid-derived suppressor cells
HCC: Hepatocellular carcinoma
MHC-1: Major Histocompatibility Complex Class I
