# Supplementary material for: Identification and validation of genes related to stem cells and telomere maintenance mechanisms as biomarkers for breast cancer
Source: Front Immunol. 2025 Jul 1;16:1618193. doi: 10.3389/fimmu.2025.1618193 (PMC12259709; doi:10.3389/fimmu.2025.1618193)
Supplement: Supplementary file 1 [file DataSheet1.pdf]

Supplementary Material

1 Supplementary Figures and Tables.

1.1 Supplementary Figures

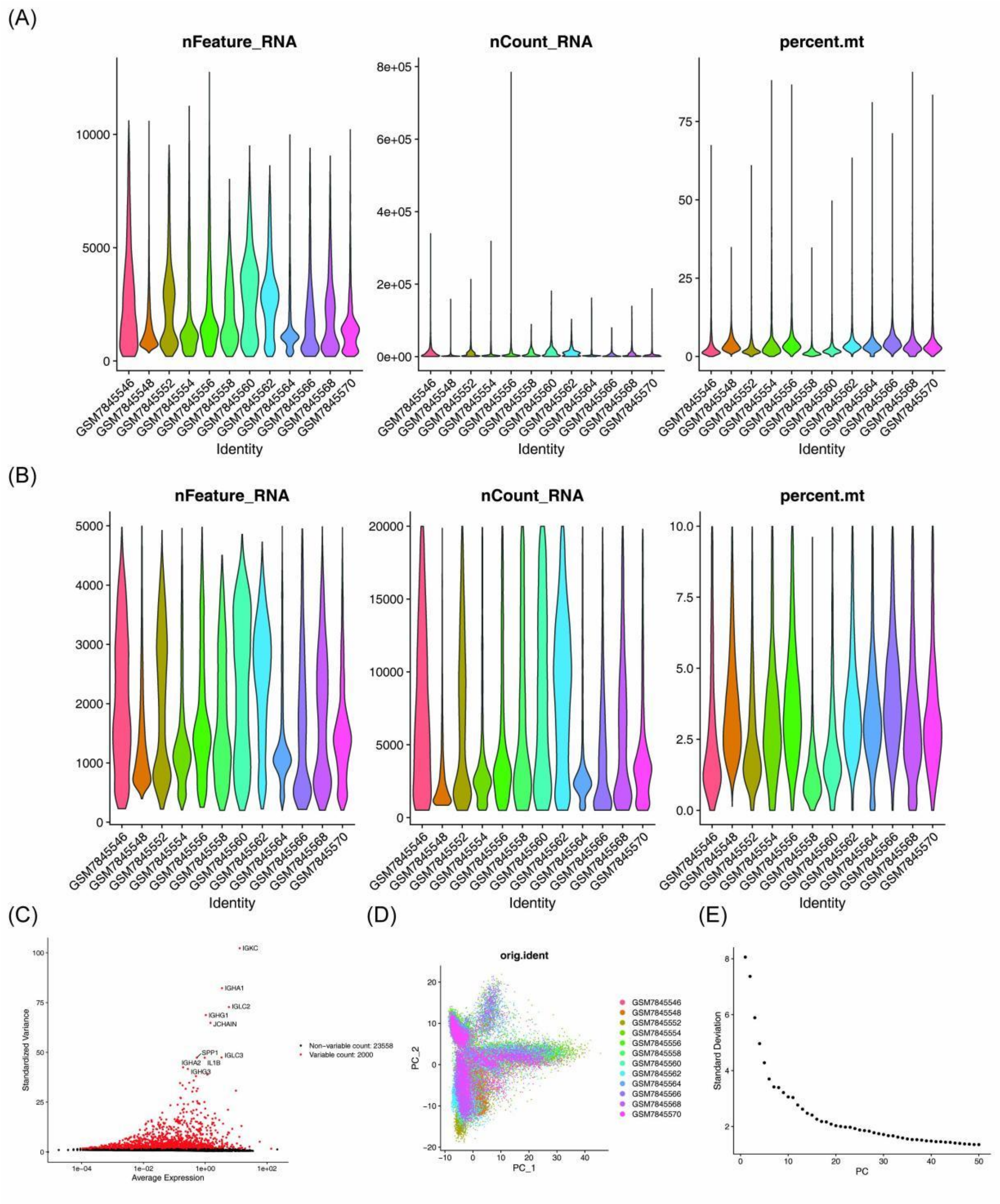

Supplementary Figure 1. Data processing in single-cell studies. (**A**) The data of single-cell sequencing before (**A**) and after (**B**) quality control. (**C**) The highly variable genes were selected after

data standardization. (**D-E**) The cell distribution (**D**) and the diagram of inflection point (**E**) according to the PCA.

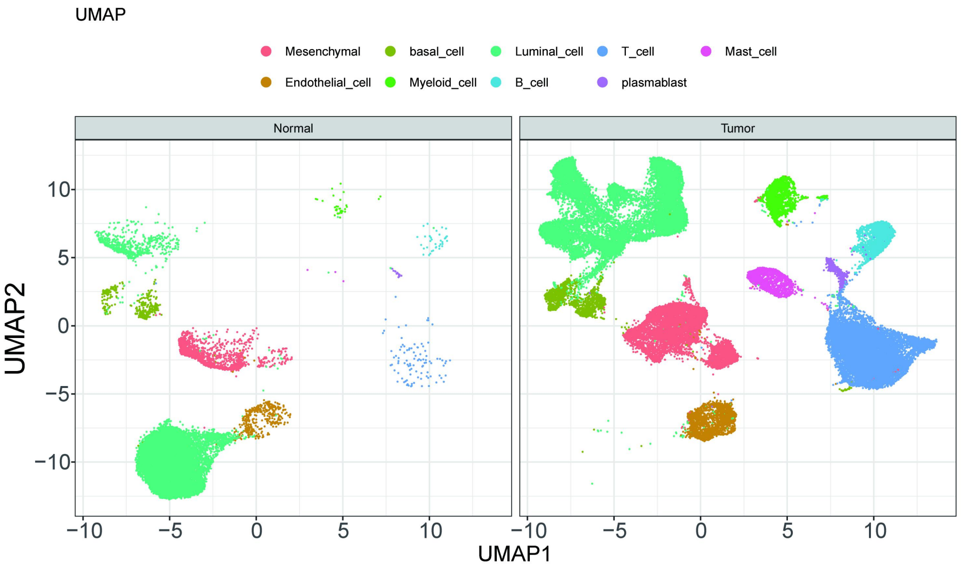

Supplementary Figure 2. The distribution of these 9 cell types in tumor and normal samples. Different colors represent different cell types.

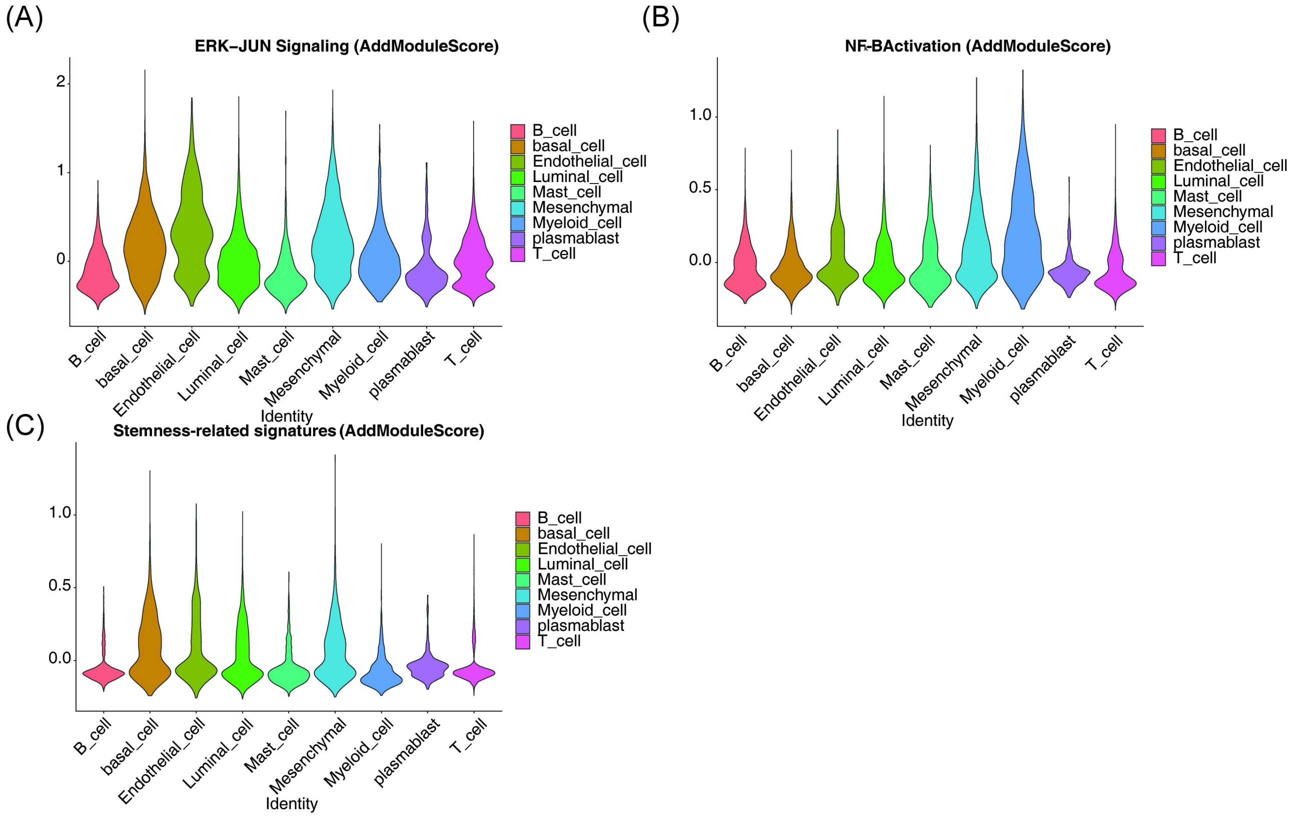

Supplementary Figure 3. Scores of various cell types on the NF-κB activation, stemness-related signatures, and ERK-JUN signaling pathways were evaluated.

1.2 Supplementary Tables

Supplementary Table 1. Primer sequences for the PCR.

Primer	Sequence
JUN F	CAGCCAGGTCGGCAGTATAG
JUN R	GGACTCTGCCACTTGTCTCC
NFKB1 F	GGTGCGGCTCATGTTTACAG
NFKB1 R	GATGGCGTCTGATACCACGG
SP1 F	CGCCCTCTGACCAAGATCACT
SP1 R	GGGAGTTGTTGCTGTTCTCATTGG
GAPDH F	CGAAGGTGGAGTCAACGGATTT
GAPDH R	ATGGGTGGAATCATATTGGAAC

Supplementary Table 2. Predictions of potential drugs for biomarkers

gene	drug	interaction score
JUN	BRUCEANTIN	2.564350169
JUN	SERGEOLIDE	2.564350169
JUN	NEOCHAMAEJASMIN A	2.564350169
JUN	HOLACANTHONE	2.564350169
JUN	IRISOLIDONE	2.564350169

NFKB1	DYRENE	0.620842673
NFKB1	BARDOXOLONE	0.620842673
NFKB1	DEHYDROXYMETHYLEPOXYQUINOMICIN	0.620842673
NFKB1	CHRYSOERIOL	0.413895115
NFKB1	ANDROGRAPHOLIDE	0.413895115
SP1	TERAMEPROCOL	58.9800539
